# Applications and Biocompatibility of Mesoporous Silica Nanocarriers in the Field of Medicine

**DOI:** 10.3389/fphar.2022.829796

**Published:** 2022-01-28

**Authors:** Chengcheng Zhang, Hongyi Xie, Zhengyan Zhang, Bingjian Wen, Hua Cao, Yan Bai, Qishi Che, Jiao Guo, Zhengquan Su

**Affiliations:** ^1^ Guangdong Engineering Research Center of Natural Products and New Drugs, Guangdong Provincial University Engineering Technology Research Center of Natural Products and Drugs, Guangdong Pharmaceutical University, Guangzhou, China; ^2^ Guangdong Metabolic Diseases Research Centre of Integrated Chinese and Western Medicine, Guangdong Pharmaceutical University, Guangzhou, China; ^3^ School of Chemistry and Chemical Engineering, Guangdong Pharmaceutical University, Zhongshan, China; ^4^ School of Public Health, Guangdong Pharmaceutical University, Guangzhou, China; ^5^ Guangzhou Rainhome Pharm & Tech Co., Ltd., Guangzhou, China

**Keywords:** mesoporous silica, MSN, cancer, targeted drug delivery, gene delivery, biodistribution

## Abstract

Mesoporous silica nanocarrier (MSN) preparations have a wide range of medical applications. Studying the biocompatibility of MSN is an important part of clinical transformation. Scientists have developed different types of mesoporous silica nanocarriers (MSNs) for different applications to realize the great potential of MSNs in the field of biomedicine, especially in tumor treatment. MSNs have achieved good results in diagnostic bioimaging, tissue engineering, cancer treatment, vaccine development, biomaterial application and diagnostics. MSNs can improve the therapeutic efficiency of drugs, introduce new drug delivery strategies, and provide advantages that traditional drugs lack. It is necessary not only to innovate MSNs but also to comprehensively understand their biological distribution. In this review, we summarize the various medical uses of MSN preparations and explore the factors that affect their distribution and biocompatibility in the body based on metabolism. Designing more reasonable therapeutic nanomedicine is an important task for the further development of the potential clinical applications of MSNs.

## Introduction

The completion of the Human Genome Project (HGP) marks the entry of human beings into the postgenome era.(Ginsburg and Phillips, 2018). The “precision medicine initiative” was proposed in the State of the Union address in 2015, and $215 million was allocated in 2016 to fund related scientific research and innovation development (The White House, 2015). In 2012, the British government announced the launch of the Genome Project, investing $523 million in cancer and rare disease research. Chinese nanomedicine is also actively being explored and has achieved good results (Zhao, 2018). Precision medicine (Adams and Petersen, 2016; Konig et al., 2017) is playing an increasingly prominent role in disease prevention, diagnosis and treatment and improving the health of the population, especially through the use of gene sequencing technology, (Ji et al., 2018; Nagahashi et al., 2019; Wakai et al., 2019), targeted drug delivery, accurate diagnostic and treatment methods, and the establishment of large-scale data for predicting health risks (Vogt et al., 2018). In practical applications, such as precision prevention, precision medicine (Bertier et al., 2016) has become the future direction of biomedicine development; compared with traditional empirical medicine, nanomedicine (Bjornmalm et al., 2017; Shi et al., 2017) can overcome medical problems based on nanotechnology. Nanomedicine integrates information about the human body and disease obtained through modern technologies, such as precision instruments and life sciences, with information obtained from traditional experience and can thus greatly reduce the uncertainty of clinical practice, improving diagnosis and treatment, especially surgical treatment. Nanomedicine (Shi et al., 2017; Bayda et al., 2019) has become a research hotspot in the international medical sciences. A variety of nanocarriers and nanomedicines have been developed, including those based on inorganic materials (such as gold (Zhang et al., 2019), iron oxide, silver (Rai et al., 2009), silica (Rotow and Bivona, 2017; Ni et al., 2018; Assaraf et al., 2019; Hu et al., 2019), and graphene) and organic nanoparticles (such as liposomes, micelles, polymers, and vesicles). Among them, mesoporous silica nanocarriers (MSNs) have many advantages, which will be discussed in detail, as follows: 1) the high specific surface area yields a high drug loading capacity for both hydrophilic and hydrophobic drugs; 2) the particle size and shape are easy to adjust to meet different drug delivery needs; 3) the easily modified active surface groups allow different coatings and targeting strategies to be applied to improve uptake, targeting and bioavailability and reduce toxic effects, thereby achieving the purpose of precision treatment and improved efficacy; 4) the mesoporous structure is ordered; MSNs with pore channels which build up an effective nano-network path enable the diffusion and extended release of drugs (Letchmanan et al., 2017). And 5) the biocompatibility is hilicic acid, are absorbed by the body or are excreted through the urinary system. It is precisely because of the above advantages that mesoporous silica has shown great prospects for applications in biomedicine and other fields. The M41S family of ordered mesoporous silica (Kresge et al., 1992) was first reported in the early 1990s. Silica-based diagnostic nanoparticles in the form of Cornell dots (C dots) have been approved by the FDA for phase I human clinical trials, (Benezra et al., 2011), which is a landmark achievement. However, for MSNs to be transformed into clinical medicines, it is necessary to understand the biological behavior and related clinical data of mesoporous silica.

**GRAPHICAL ABSTRACT:**
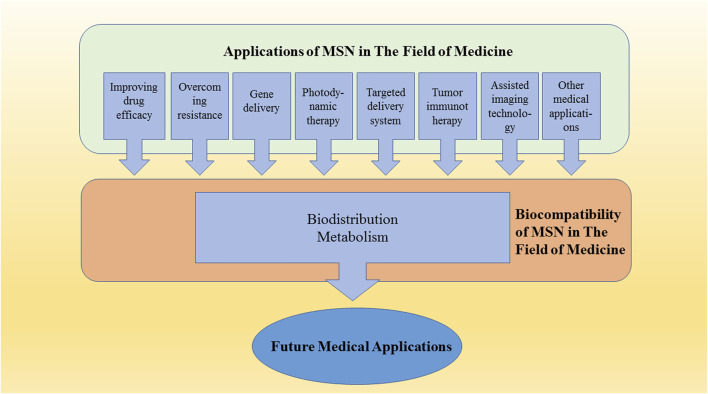
Mesoporous silica nanocarrier (MSN) preparations have a wide range of medical applications. Studying the biocompatibility of MSN is an important part of clinical transformation.

The mechanism of interaction between nanoparticles and various cells and tissues in the human body is the key to determining the medical applicability of nanotechnology. Compared with soluble molecules and drugs, the delivery of drugs using nanocarriers is more complicated because the behavior of nanocarriers is related not only to the molecular weight, solubility, and physical and chemical properties of the loaded drugs but also to the pore size distribution, and particle size of the nanocarrier. Coatings, targeting groups and other factors are also closely related. Therefore, for drug delivery using nanocarriers, it is necessary to detect, understand and optimize the release parameters, distribution, metabolism, excretion and biocompatibility of the drugs in the body for optimal clinical results. These are the current challenges in nanomedicine.

In this review, we summarize the current reliable research data and describe the application of mesoporous silica as a nanocarrier in gene therapy, photodynamic therapy, molecular targeted drug delivery, vaccine delivery, and imaging, among others. MSN distribution and metabolism are discussed in detail. The importance of biodistribution and biocompatibility is explained, and key points of future research are discussed.

## Applications in Medicine

**FIGURE 1:**
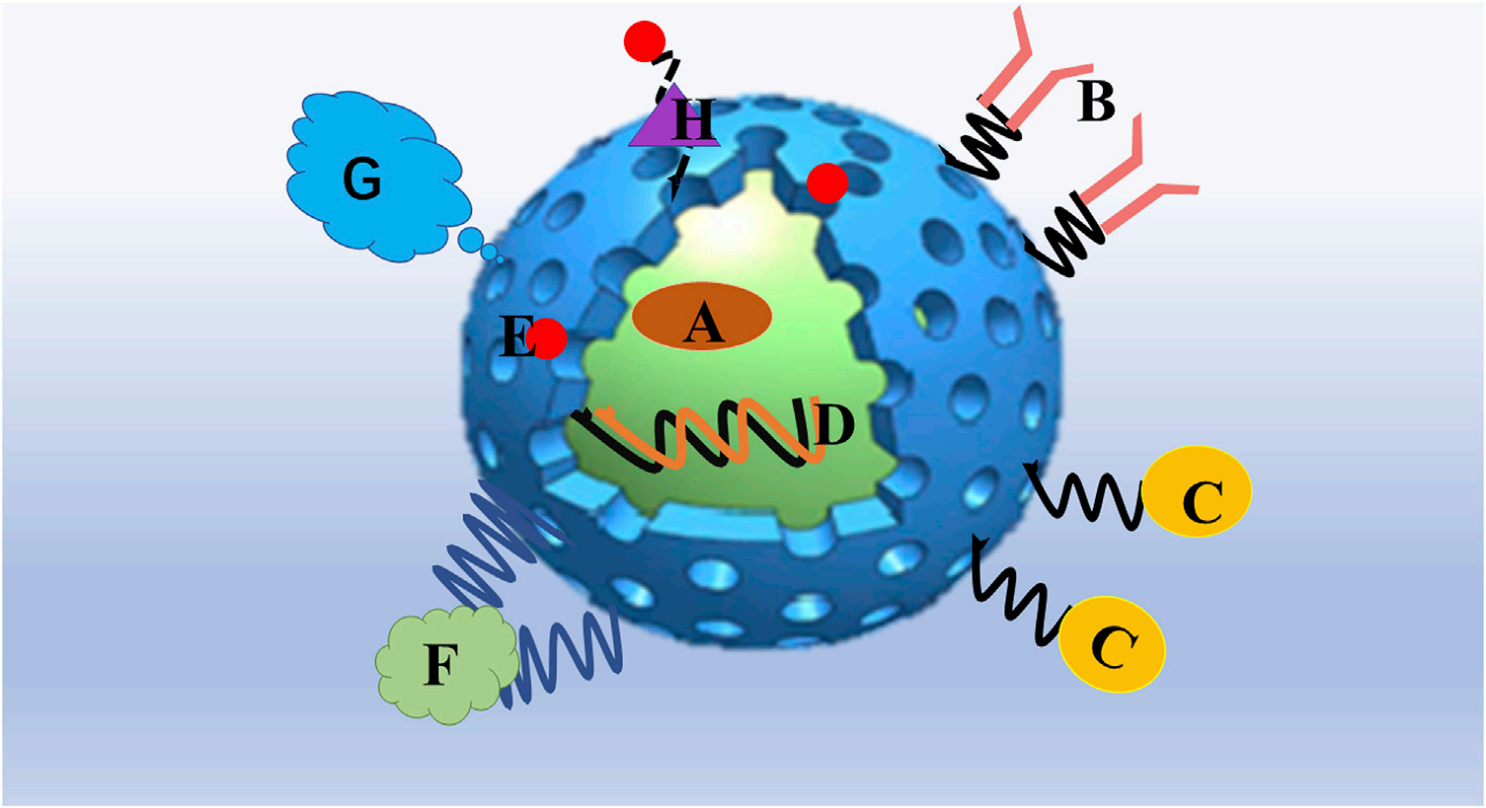
Mesoporous silica nanoparticles are particularly promising as a platform for drug delivery. **(A)** Hydrophobic/hydrophilic drug and combining drugs could be entrapped in the interior of the MSN. **(B)** Targeting ligands such as antibodies. **(C)** Bioimaging agents such as magnetic nanoparticles, quantum dots. or fluorophores. **(D)** gene therapy agents such as plasmids, DNA, small interfering RNA (siRNA), micro RNA (miRNA), and short-hairpin RNA (shRNA). **(E)** nanoparticles attached to MSNs as functional gatekeepers. **(F)** Stimuli-responsive polymers. **(G)** Grafting with a protecting polymer, such as PEG, shields the MSN surface from interacting with opsonizing proteins. **(H)** Stimuli-responsive linkers, which chemically attach MSNs and gatekeepers.

MSNs have the advantages of an ordered mesoporous structure, an adjustable pore size, a high specific surface area, and easily modified active surface groups (Baek et al., 2015). MSNs are particularly promising as nanodrug carriers; biomedical applications require MSNs to maintain a high degree of dispersion and colloidal stability. Aggregation affects MSN internalization by cells, which increases the difficulty of controlling the biodistribution; furthermore, larger particle sizes caused by agglomeration may lead to increased biological toxicity. Based on the easy-to-modify hydroxyl groups on the MSN surface, chemical modifications, such as with a protein, polymer coating, or phospholipid bilayer, can be used to maintain its colloidal stability and reduce the occurrence of agglomeration. (Figure 1). Various MSNs have been developed for targeted drug, gene, and protein delivery, along with composite nanomedicines for diagnostic bioimaging, tissue engineering, cancer treatment, vaccine development, biomaterial applications, and theranostics (Singh et al., 2017); (Table 1)

**TABLE 1:** Applications and biocompatibility of MSNs.

Nanomedicine	Purpose of treatment	Nanodrug size	Cargo	Modification	Modification function	Citation
MSNs-DOX@PDA-PEG	Improving the efficacy and reducing the side effects of anticancer drugs	198 nm	DOX	PEG-PDA	PEG increases the stability and biocompatibility; PDA functions as a pH-sensitive gatekeeper	Duo et al. (2017)
LM@MSNs/DOX@HA	Inhibiting solid tumor growth under near-infrared (NIR) irradiation by synergistic photothermal therapy/chemotherapy	160 nm	DOX	Liquid metal HA	Synergistic photothermal therapy/chemotherapy	Hu et al. (2019)
M-MSNs-DOX	Improving the efficacy and reducing the side effects of anticancer drugs	200 nm	DOX	PEG	PEG increases the stability and biocompatibility	Shao et al. (2016)
H-MSNs-DOX/siRNA	Inhibiting MDR tumor growth	70 nm	DOX/siRNA	—	—	Sun et al. (2017)
PTX/GEM LB-MSNPs	Synergistically suppressing pancreatic cancer stromal volume and tumor size	112 nm	GEM/PTX	Lipid-coated	Facilitate coentrapment of hydrophobic drugs	Meng et al. (2015)
PTX/TET-CTAB@MSNs	Combining drugs for antitumor activity and the reversal of MDR activities	125 nm	PTX/TET	CTAB	pH-responsive release property	Jia et al. (2015)
PMSN-PEI-CQ	Highly efficient transfection of plasmid DNA and reducing cytotoxicity	174.5–215 nm	CQ; pDNA	PEI	Protect the pDNA from nuclease degradation	Zarei et al. (2018)
MSN-2NH_2_/CpG	CpG oligodeoxynucleotide delivery	178 nm	CpG ODN	NH _2_ -TES, 2NH _2_ -TES, 3NH _2_ -TES	Larger loading capacity, significantly enhance the serum stability of CpG ODN	Xu et al. (2015)
MSNs-NH_2_/dsDNA	Enhancing the delivery efficiency of immunostimulatory DNA drugs	190 nm	dsDNA	-NH2	Higher efficiency of cell uptake	Tao et al. (2014)
MSNPs-PEI-DOX/MDR1-siRNA	MDR cancer	150 nm	DOX MDR1-siRNA	PEI	Efficient transfection into KBV cells	Wang et al. (2018)
PEG-PEI@MSNs@siRNA	siRNA delivery	113 nm	siRNA	PEI-PEG	Good synthesis reproducibility and scalability	Ngamcherdtrakul et al. (2018)
KIT-6-MSNs@ siRNA	High nucleic acid loading capacity	200–400 nm	siRNA	—	—	Meka et al. (2016)
LPMSNs@TRAF3-shRNA	Inhibiting the mRNA and protein expression of TRAF3	170 nm	shRNA-TRAF3	—	—	Zhang J. et al. (2016)
MONs–PTAT@pDNA	Highly efficient intranuclear gene delivery	160 nm	pDNA	PTAT	High loading capacity, improved protection for the loaded gene, enhanced transfection efficiencies of EGFP plasmid	Wu et al. (2015)
CP-MSNPs@siRNA	Delivering siRNA for cancer therapeutics	105 nm	siRNA	CP	Positive charge for the loading of siRNA	Shen J. et al. (2014)
CM/SLN/Ce6	Tumor-targeted PDT of gastric cancer	115 nm	Ce6	Cellular membrane (CM)	High biocompatibility and inheritance of the merits of the source cells	Yang et al. (2019)
AuNRs@MSNs-RLA/CS(DMA)-PEG	Enhancing photodynamic and photothermal tumor therapy	200 nm	ICG AuNR	RLA/CS(DMA)-PEG	Tumor targeting and pH response	Liu et al. (2018)
^64^Cu-HMSN-ZW800-TRC105	Tumor-targeted positron emission tomography (PET)/near-infrared fluorescence (NIRF) dual-modality imaging	150 nm	^64^Cu	TRC105	Target tumor vasculature	Chen et al. (2014)
YSPMOs(DOX)@CuS	Multifunctional triple-responsive platform for chemo-photothermal therapy	222.6 nm	DOX	CuS	Avoid premature leakage in the delivery process, provide the photothermal therapy (PTT) ability	Cheng et al. (2018)
HmSiO_2_-FA-CuS-PEG/DOX	Nanoplatform for targeted chemo-photothermal therapy	155 nm	DOX	FA CuS	Target cancer cells Chemo-photothermal therapy	Liu et al. (2014)
PSiNPs@ PELA-PEG	Synergistic effects and MDR inhibition	286 nm	Afatinib, rapamycin, docetaxel	PELA-PEG	Achieve high biocompatibility and low permeability	Zhang et al. (2019)
CuS@MSNs-TRC105	Photothermal ablation properties and tumor vasculature targeting	65 nm	CuS	TRC105	Target tumor vasculature	Chen et al. (2015)
MSNP-CYS-5FU-FA-BA@DOX-CD	Augmented the innate and adaptive immune defense mechanisms, Significantly reduced the tumor load and enhanced the survival of the animals	110 nm	Dox; 5-FU	FA	Active targeting by folic acid directs drugs in the close proximities of the tumor cells, causing efficient killing and significant growth inhibition	Srivastava et al. (2020)
Ru@MSNs	Exhibited high *in vivo* antitumor activity, the nanosystems at 20 nm exhibited low toxicity, the larger (80 nm) showed superior potential for overcoming MDR.	20 nm, 40nm, 80 nm	Ru	FA	Facilitate selectivity toward hepatocellular carcinoma cells	(Tang et al., 2013; Ma et al., 2018)
DTX-Lac-MSN	A hepatoma-targeting drug delivery system	100 nm	DTX	Lactose	Specifically target ASGPR	Quan et al. (2015)
MSNs-FA-Q	Targeted delivery with enhanced bioavailability	200 nm	Quercetin	FA	Target breast cancer cells	Sarkar et al. (2016)
MSNs-FA-TAN-MB	Ultrasound response property, tumor targeting and imaging in tumor therapy	2,608 nm	Tanshinone IIA (TAN)	FA MB	Tumor targeting, high biocompatibility	Lv et al. (2017)
MSR-MSNs	Dual-scale vaccine transport into host dendritic cells (DCs) to enhance cancer immunotherapy	150 nm	OVA, CpG-ODNs	—	—	Nguyen et al. (2020)
Trp2@HMSNs	Improved the antigen-loading efficacy, sustained drug release profiles, enhanced the phagocytosis efficiency, enabled DCs maturation and Th1 immunity, sustained immunological memory, and enhanced ​the adjuvant effect	200 nm	Trp2	PEI	Acted as an etching agent, protecting agent, soft template, and promoter	Liu et al. (2019)
LB-MSNs-OVA	Intradermal antigen delivery system	213 nm	OVA	Lipid bilayer	Significantly improve the colloidal stability and reduce the premature release of OVA	Tu et al. (2017)
Gd@SiO_2_-DOX/ICG-PDC	Cancer treatment and magnetic resonance imaging	214 nm	DOX, ICG Gd(III)	PDC	Protect from quick release of drugs and increase cellular uptake	Cao et al. (2015)
MSNs-DOX-Ag_2_Se	Chemo-photothermal therapy	130 nm	DOX	Ag_2_Se QD	Enhance photothermal properties and act as “gatekeepers"	Li et al. (2019)
Apt-PTPA-MSHNs	Highly efficient MRI contrast agents	200 nm	PTPA	EpCAM	Anti-EpCAM aptamer was conjugated with epoxy-functionalized PTPA MSHNs to improve selectivity toward the cancerous cells	Dineshkumar et al. (2019)
Mn-DTPA-MSNSs	Liver-specific positive MRI contrast agent	116 nm	—	Mn^2+^	MRI contrast agent	Pálmai et al. (2017)
Fe_3_O_4_@mSiO_2_/PDDA/BSA-Gd_2_O_3_	T_1_-T_2_ molecular magnetic resonance imaging of renal carcinoma cells	345 nm	BSA-Gd_2_O_3_, Fe_3_O_4_	AS1411	Specifically combine with nucleolin on the surface of the tumor cell	Li et al. (2018)
MSNs-GTMC-PMMA	Functionalization for orthopedic surgery to prevent post-surgery infection	100–400 nm	GTMC	PMMA	Critical weight-bearing mechanical properties	Letchmanan et al. (2017)
GTMC/TBMC/MSN/Simplex-P	The combination of excellent mechanical properties and sustainable drug delivery efficiency demonstrates the potential applicability for orthopedic surgery to prevent post-surgery infection	400 nm	GTMC TBMC	PMMA	Critical weight-bearing mechanical properties, bending modulus and compression strength of bone cement	Letchmanan et al. (2017)
SiO_2_-PMMA	Mimicking the mechanical properties of human enamel and hardness compatibility with human enamel	7 nm	—	PMMA	Achieve hardness compatible with that of human enamel and an elastic modulus similar to that of human dentin	Ikeda et al. (2019)
PDG-MSNPs	Improved the engraftment of islets (i.e., enhanced revascularization and reduced inflammation), re-establishment of glycemic control	120 nm	Glutamine	Polydopa-mine	Resulted in a delay in the release of glutamine	Razavi et al. (2020)
OST-MSNs-PA@PEI-siRNA	Increase expression of osteogenic related genes improving the bone microarchitecture	100 nm	Osteostatin SOST siRNA	alendronate (ALN) modified PEG	Confer the nanoparticles good colloidal stability and bone targeting capacity	Mora-Raimundo et al. (2021)
Ag@Vm-ge	Combined with the gentamicin delivery, the pathogenic bacteria in diabetic wound can be completely eradicated	145 nm	Gentamicin	—	—	Wang et al. (2021)
colchicine MSNs/chitosan-pullulan hydrogel	Enhanced the drug skin permeation and therapeutic activity in comparison to conventional free colchicine	167.1 ± 51.36 nm	Colchicine	Carboxyethyl chitosan/oxidized pullulan	Efficient transdermal delivery	Mohamed et al. (2020)
Ce@MSNs	Stimulated osteoblast cells to produce bone matrix and demonstrated antioxidant properties in a co-culture cells without osteogenic supplements	80 nm	Ce	—	—	Pinna et al. (2021)

### Improving the Efficacy and Reducing the Side Effects of Anticancer Drugs

At present, chemotherapy is one of the most effective methods for the treatment of cancer, and chemotherapy, surgery and radiotherapy together form the three pillars of cancer treatment. In chemotherapy, drugs are used to kill cancer cells to achieve the goal of treatment. However, whether is the drugs are taken by mouth, injection, intraperitoneal injection, or other routes, they will enter the systemic circulation and cause uncontrollable side effects. MSNs have a high drug loading capacity and can be loaded with small-molecule drugs, especially hydrophobic drugs; at the same time, this approach can prevent drug degradation and physiological toxicity to healthy tissues caused by premature drug exposure. Thus, MSNs are of great significance for the delivery of hydrophobic anticancer drugs, drugs with narrow therapeutic windows, and chemotherapeutics with significant side effects. Drugs with narrow therapeutic windows is also called narrow therapeutic index (NTI). NTI drugs can be defined based on the steepness of the dose–response relationship and the degree of overlap between the effective and the toxic concentrations. MSNs are good tool about solving the problem of NTI, because they could improve the efficacy and reduce the side effects of drugs (Zenych et al., 2020; Habet, 2021). Duo et al (Duo et al., 2017) designed the doxorubicin (DOX)-loaded mesoporous silica nanomedicine MSN-DOX@PDA-PEG for the treatment of breast cancer; compared with free DOX, the nanomedicine showed a higher cell uptake efficiency in MCF-7 and MDA cells. The cytotoxicity test in MB-231 cells proved that the nanomedicine had almost no cytotoxicity. *In vivo* experiments showed that the smallest tumor volume and greater anticancer efficacy in the mesoporous silica nanomedicine group; additionally, body weight monitoring and hematoxylin and eosin (H&E) staining of organ (including heart, liver, spleen, lung and kidney) tissue sections showed no histopathological abnormalities, indicating MSN-DOX@PDA-PEG has good biocompatibility and low *in vivo* toxicity.

Mesoporous silica nanomedicine can not only improve the therapeutic effect of the anticancer drug DOX but also reduce its side effects. Hu et al. (Hu et al., 2019) designed a DOX-loaded MSN (LM@MSN) via surface modification and liquid metal immobilization for cancer treatment; the near-infrared (NIR) dye indocyanine green (ICG) was encapsulated in the nanosystem. *In vivo* fluorescence imaging was performed to evaluate its targeting performance. The results of *in vitro* fluorescence imaging showed aggregation of the targeted drug in the tumor. After 36 h, the content of heparin in the tumor was higher than that in the heart, spleen, kidney and other organs. When tested in tumor cells (4T1, HeLa, MCF-7) and normal cells (3T3), the normal cells showed a viability of greater than 80%, demonstrating low toxicity and good activity even at relatively high concentrations, in turn indicating that this MSN has good biocompatibility. Shao et al (Shao et al., 2016) designed a “nanobullet” consisting of magnetic Fe_3_O_4_ as the head and DOX-loaded MSN as the body (M-MSN-DOX); this multifunctional nanocomposite material showed excellent targeting, with greater uptake by HepG2 tumor cells. The administration of DOX and M-MSN-DOX via a tail vein injection for 16 days effectively inhibited the growth of orthotopic liver tumors and the deterioration of other analyzed liver tumor mouse model parameters, including body weight, blood chemistry and major organ and tissue toxicological indicators. No pathological damage was observed in mouse brain, heart, liver, spleen, lung or kidney tissue sections, indicating that this mesoporous silica nanomedicine not only has good biocompatibility but is also effective for improving the efficacy and reducing the side effects of the drug.

### Combining Drugs and Overcoming Resistance

**FIGURE 2:**
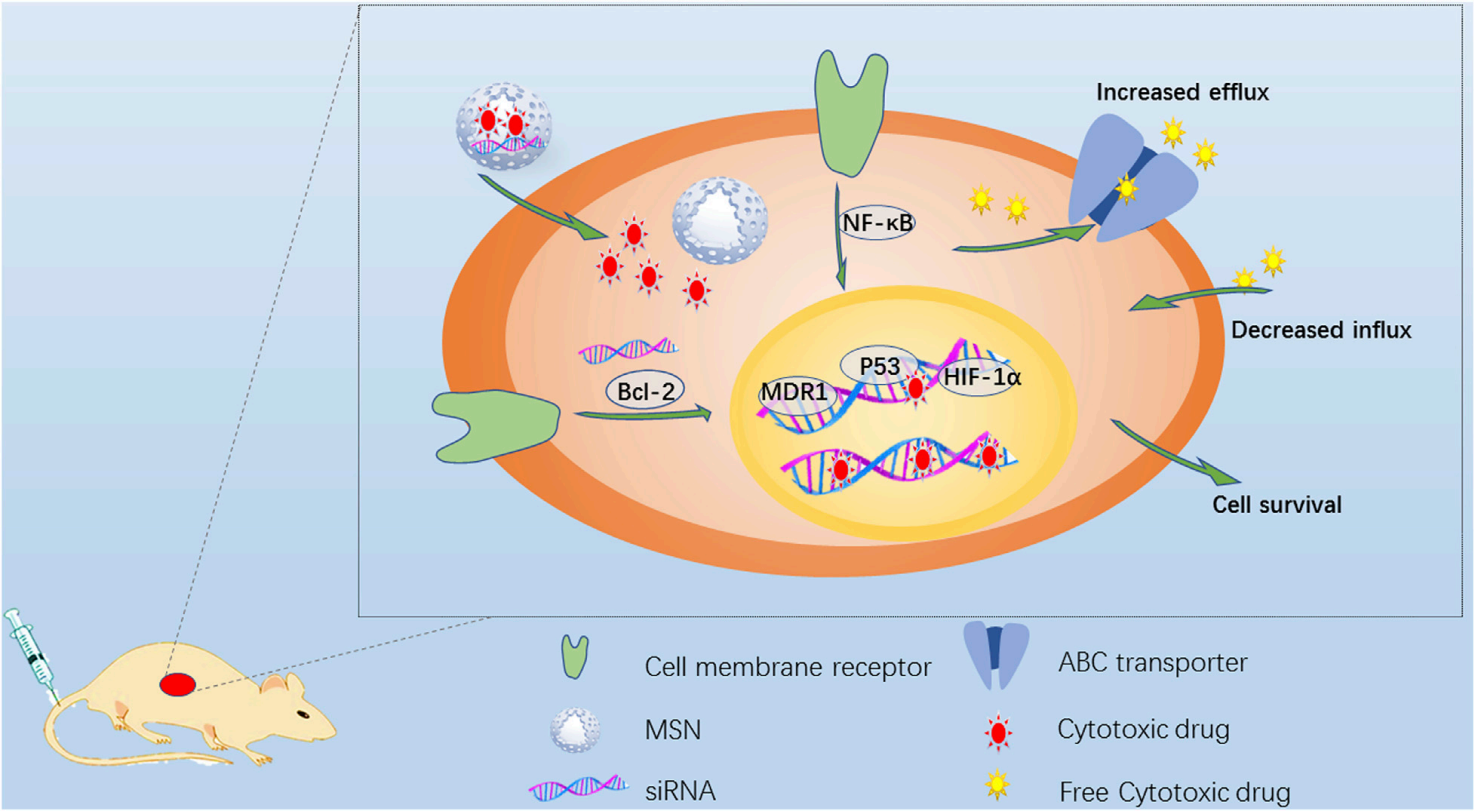
Schematic diagram of strategies to overcome MDR. Drug-resistant cancer cells have many ways to escape cytotoxic drugs, including the overexpression of ABC transporter to increase drug efflux, repair DNA and inhibit drug-induced apoptosis. The anti-apoptotic signal transduction pathway always involves the activation of Bcl-2, MDR1, NF-κB and HIF-1 overexpression and the mutation of tumor suppressor gene p53. MSN is endocytosed into drug-resistant cancer cells, and the loaded cytotoxic drugs and/or siRNA targeting related genes can be continuously released to reduce drug resistance.

The current important standard for cancer treatment (Yardley, 2013) is based on increasing the dosage and scheduling intensity of therapeutic drugs to avoid chemotherapy resistance. Multidrug resistance (MDR) (Rotow and Bivona, 2017; Fang et al., 2018; Assaraf et al., 2019) is the main obstacle to cancer chemotherapy; MDR can severely hinder the efficacy of anticancer drugs and even lead to treatment failure. The drug resistance mechanism of tumor tissue is very complex and involves multiple dynamic mechanisms. MDR can usually be divided into two categories. Drug efflux pump (P-glycoprotein (Thorat et al., 2020) and multidrug resistance protein MRP1) overexpression is an effective method (Figure 2).

Drug transport pumps can actively prevent the treatment of tumors. The drug is pumped out of the cell, increasing the efflux of the drug and reducing the accumulation of the drug in the cell. The second category mainly refers to the activation of cell antiapoptotic defense pathways, such as by drug-induced B-cell lymphoma 2 (BCL-2), inhibitor of apoptosis protein (IAP) and FLIP protein expression, leading to reduced drug sensitivity. These two mechanisms are sometimes active at the same time. In view of the unique properties of mesoporous silica, different drug delivery strategies have been designed to overcome drug resistance. The energy-dependent endocytosis of MSNs promotes cell absorption, improves drug efficacy and bypasses drug efflux pumps to overcome drug resistance. Sun et al. (Sun et al., 2017) designed a unique core-shell-level mesoporous silica/silicone nanosystem. The small mesopores in the core were loaded with small-molecule drugs, and the large mesopores in the shell were integrated with siRNA by disulfide bonds. During treatment, the reactivity of the disulfide bonds in the shell to the tumor microenvironment (TME) caused them to break down, first releasing the siRNA to inhibit the expression of P-gp to inhibit MDR and then releasing the small-molecule drug DOX from the core to exert a therapeutic effect. *In vivo* experiments showed that the tumor inhibition rate in the free DOX group was 50.7%, while that in the H-MSN-DOX/siRNA group reached 87%, demonstrating a significant inhibitory effect on drug resistance. At the same time, mice in the free DOX group lost weight, but those in the other groups, including the H-MSN-DOX/siRNA group, showed normal weight changes. Compared with the injection of free drug, administration of the H-MSN-DOX/siRNA delivery system greatly enhanced the therapeutic efficacy while significantly reducing the side effects of the delivered anticancer drug. MSNs loaded with different drugs may overcome the problem of drug resistance by strategically improving the therapeutic efficacy of the drugs. Meng et al (Meng et al., 2015) designed a mesoporous silica nanoparticle (MSNP) carrier to jointly deliver paclitaxel (PTX) and gemcitabine (GEM) for the synergistic treatment of pancreatic cancer. The codelivery of PTX/GEM by the LB-MSNP yielded better results than the delivery of free GEM and Abraxane in xenotransplantation and orthotopic tumor animal models, with a therapeutic effect equivalent to that of 12 times the dose of Abraxane. Similarly, Jia et al (Jia et al., 2015) designed multifunctional MSNPs loaded with the antitumor drug PTX and the multidrug resistance reversal agent tetrandrine (TET). The results of experiments in MCF-7 human breast cancer cells and MCF-7/ADR multidrug-resistant cells showed that the PTX/TET-CTAB@MSNs significantly inhibited the proliferation of the drug-resistant cells and completely reversed the resistance to PTX.

The combination of medications can improve the therapeutic effect of the drugs; additionally, different drugs can be selected for loading according to different treatment strategies and individual differences. This approach can reduce the side effects of anticancer drugs and avoid the development of drug resistance.

### MSNs for Gene Delivery

Gene therapy has broad prospects, but naked nucleic acids are not easily internalized by cells; they have poor biological stability and a short half-life and are subject to intracellular degradation prior to entering the nucleus. The systemic delivery of carrier particles can overcome these problems. At present, gene delivery systems can be divided into two categories: viral vectors and nonviral vectors. Although viral vectors have advantages in delivering genes, they have potential safety hazards. Nonviral vectors have been a research hotspot in recent years, including polymers, recombinant proteins, cationic compounds and inorganic nanoparticles. However, cationic materials are usually associated with high toxicity, recombinant proteins show low cost performance, and liposomes can enable effective gene transfection but are unstable. Compared with other nanoparticles, inorganic nanoparticles are simple to prepare and easy to functionalize and have good biocompatibility and excellent physical and chemical stability. MSNs have favorable characteristics for gene delivery, including a narrow pore size distribution and the ability to can effectively protect the cargo up to the release point, and the surface chemical structure facilitates the optimization of adsorption and release characteristics. Thus, MSNs are considered promising carriers for gene therapy.

**FIGURE 3:**
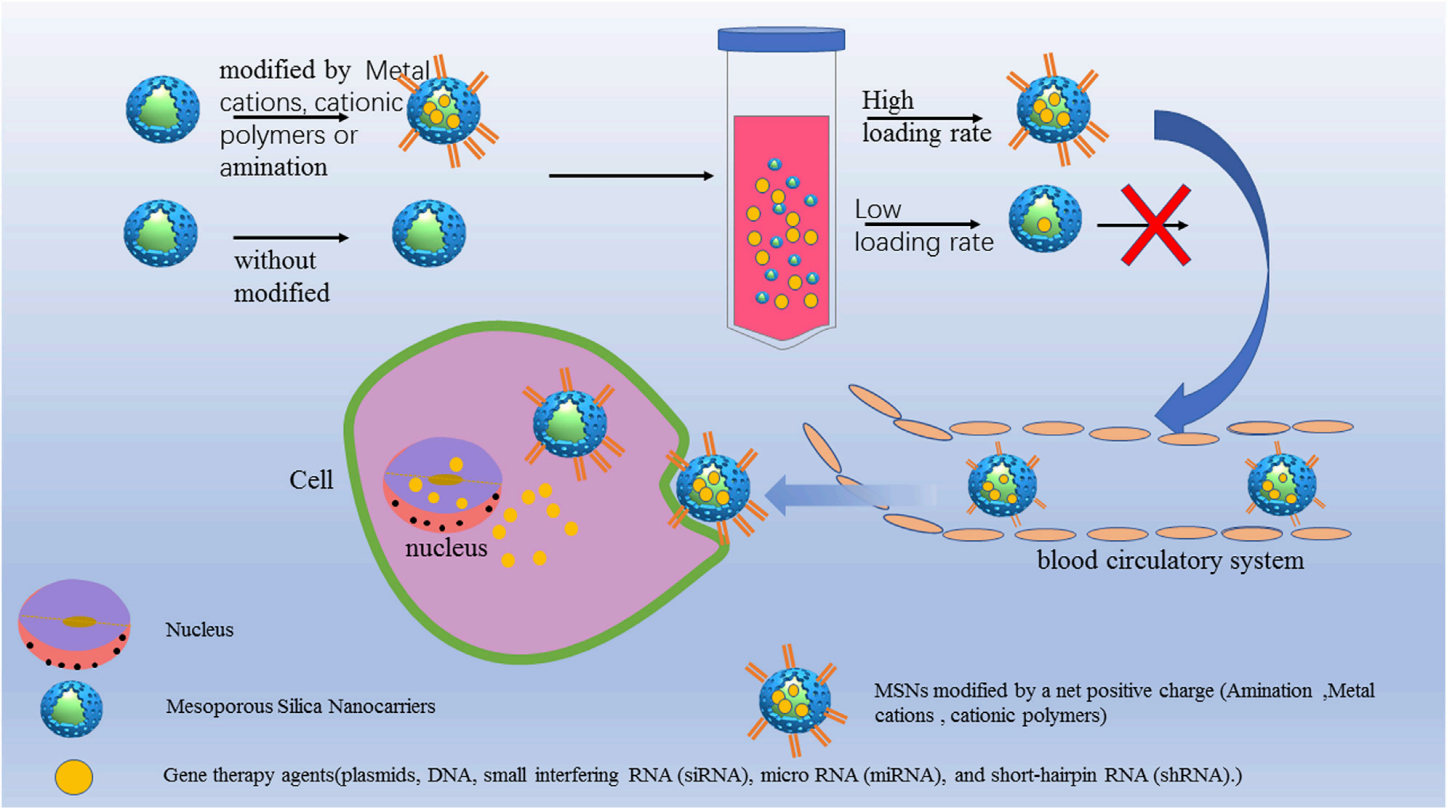
MSNs for gene therapy to increase the cell uptake and transfection efficiency. MSNs modified by a net positive charge such as amination modification, metal cations and cationic polymers. The aminated MSN delivery systems had a higher loading capacity and showed greater delivery efficiency and protection, resulting in significantly improved serum stability without cytotoxicity.

The current methods to improve the performance of mesoporous silica-loaded genes are as follows: 1) positive charge functionalization, such as by amination (Cheang et al., 2012; Tao et al., 2014; Xu et al., 2015; Zhang M. et al., 2016) and cationic polymer (Ngamcherdtrakul et al., 2018; Wang et al., 2018) functionalization, yielding modified MSNs with a net positive charge that promotes gene loading by enhancing electrostatic interactions with nucleic acids; (Figure 3); 2) synthesizing large pore size MSNs to realize improved gene protection and transportation. (Wu et al., 2015; Zhang J. et al., 2016; Meka et al., 2016).

To study the effect of amination on the delivery efficiency of nucleic acids, Xu et al.(Xu et al., 2015) modified MSNs with NH2-TES, 2NH2-TES and 3NH2-TES and loaded them with CpG oligodeoxynucleotide (CpG ODN) to form MSN-NH2/CpG, MSN-2NH2/CpG, MSN-3NH2/CpG complexes. The aminated MSN delivery systems had a higher loading capacity and showed greater delivery efficiency and protection, resulting in significantly improved serum stability without cytotoxicity. Similarly, the modification of mesoporous silica with cationic polymers can also improve the gene loading performance. Shen et al (Shen J. et al., 2014) designed a universal siRNA carrier composed of MSNPs functionalized with polyethyleneimine (PEI) grafted with cyclodextrin (CP), allowing the positive charge loading and release of siRNA *in vivo* through electrostatic interaction. Compared with free siRNA, the CP-MSNPs reduced the intracellular degradation rate of siRNA by 47.4%, and the cell internalization rate reached 99.2% within 24 h. *In vivo* microscopy monitoring in a tumor mouse model showed that the maximum concentration at the tumor site could be reached in 20 min, targeting and effectively inhibiting the gene expression of glycolytic pyruvate kinase isoenzyme (PKM2). Wang et al (Wang et al., 2018) designed MSNs carrying MDR1-siRNA to block the expression of MDR1 and loaded them with the anticancer drug DOX. The cationic polymer PEI was used to modify the MSNs to obtain a positive charge for better MDR1-siRNA loading. Meka et al. (Meka et al., 2016) prepared MSNs with 9 nm mesopores. The hydrophobic octadecyl-conjugated MSNs showed a high loading capacity and effectively delivered siRNA to cancer cells, inhibiting cancer cell proliferation. Wu et al (Wu et al., 2015) successfully developed mesoporous organosilica nanoparticles (MONs) with cell-penetrating peptides (TAT) for efficient nuclear gene delivery with a high drug loading capacity, which resulted in better loaded gene protection and improved EGFP plasmid transfection efficiency.

At present, surface amino and cationic modification as well as large-pore-size mesoporous silica can effectively improve gene delivery and have broad prospects for application in cancer therapy drug/gene delivery.

### Application of Mesoporous Silica in Photo-Triggered Therapy

Both photothermal therapy (PTT) and photodynamic therapy (PDT) are important class of therapy approach by MSNs, have been demonstrated the priorities to elevate cancer therapeutic efficacies and diminish undesired side effects through different mechanisms in cancer treatment. Photothermal therapy (PTT) (Shen et al., 2016) is a minimally invasive method selectively ablate cancer cells by converting light energy into heat. It has become a promising alternative to radiotherapy and chemotherapy. The basic principle of PDT is that the irradiation of photosensitizers at specific wavelengths causes cells to produce create cytotoxic singlet oxygen or ROS, leading to cell death (Singh et al., 2017; Liang et al., 2020; Wang et al., 2020).

To improve the therapeutic effect, synergistic effects between photothermal agents and antitumor drugs (Youssef et al., 2018; Hu et al., 2019; Yang et al., 2019; Zhang et al., 2019) have become a new hot topic of research. Many dosage forms of DOX-loaded mesoporous silica for cancer treatment have been developed and achieved good results. Hu et al (Hu et al., 2019) designed immobilized liquid metal nanoparticles based on a surface mesoporous silica coating strategy (LM@MSNs) and loaded them with the anticancer drug DOX and decorated them with hyaluronic acid (HA), resulting in the construction of the tumor-targeted nanomedicine LM@MSN/DOX@HA. Under NIR irradiation, the immobilized LM nanoparticles exerted a photothermal effect and a synergist effect with the anticancer drug to kill cancer cells. HA helped the nanocarrier achieve targeted recognition and reduced its toxic side effects on normal tissues. Similarly, Liu et al (Liu et al., 2018) designed a multifunctional nanoplatform based on mesoporous silica-coated gold nanorods (AuNRs@MSNs) that were loaded with ICG and RLA peptide with plasma membrane permeability and a mitochondrial targeting ability ([RLARLAR]2) and capped with β-cyclodextrin (β-CD). The weakly acidic microenvironment of tumor tissue induced polymer dissociation and re-exposure of the RLA peptide, promoting targeted accumulation of the nanocarrier in mitochondria. When irradiated with an 800 nm laser, the local electric field enhancement of the AuNRs significantly increased the ROS production of ICG, yielding a photothermal effect. The production of ROS and local hyperthermia led to mitochondrial dysfunction, leading to obvious tumor cell apoptosis and necrosis. Zhang et al (Zhang et al., 2019) used microfluidic technology to encapsulate mesoporous silica and AuNRs in a hybrid polymer body to construct a composite material. The hybrid polymer was composed of a combination of poly (ethylene glycol)-b-poly (lactic acid) diblock copolymer and phospholipids, resulting in high biocompatibility and low permeability. This material could be used to realize combined antitumor, photothermal, and targeted therapy.

Copper sulfide (CuS) nanoparticles have the characteristics of a low synthesis cost, a wide NIR absorption range, good biocompatibility and a good NIR photothermal conversion efficiency. An increasing number of nanodrugs based on the photothermal effect of CuS are being developed (Chen et al., 2014; Cheng et al., 2018). Liu et al. (Liu et al., 2014) developed hollow MSNPs loaded with CuS as a photosensitizer, DOX as an anticancer drug, and folic acid (FA) as a targeting agent. The system showed a good photothermal effect and an excellent DOX loading capacity. It enters cancer cells through a targeted receptor-mediated endocytic pathway; the change in pH and exposure to NIR irradiation trigger the release of DOX, driving the synergy of chemotherapy and PTT.

Wang et al. (Wang et al., 2020)reported a redox nanocarrier (called RN) is prepared by hollow mesoporous silica sphere (HMSNs) and a redox-responsive polymer ligand. The nanocarrier is loaded with catalase, metformin, and photosensitizer chlorin e6 (Ce6). With optimal size, redox-responsive drug release behavior and excellent singlet oxygen production, the RN have potential to enhance anti-tumor efficiency. The *in vitro* chemo-photodynamic synergetic experiments indicated that the NPs had excellent reactive oxygen species generation and remarkable cancer cell killing efficiency under laser irradiation.

The critical challenges in photodynamic and photothermal chemotherapies of cancer are the limited penetration depth of light, instability of photosensitizers, and non-degradable character of inorganic nanomaterials (Cheng et al., 2020; Wang et al., 2020). Improving the photothermal conversion efficiency and reducing the laser power density have become the directions of PTT development. Combination photo-chemotherapy, including photothermal-chemotherapy, photodynamic-chemotherapy, and photodynamic-photothermal-chemotherapy, has demonstrated the priorities to elevate cancer therapeutic efficacies and diminish undesired side effects through different mechanisms in cancer treatment.

### Targeted Delivery System Based on Mesoporous Silica

Targeted drug delivery systems (TDDSs) (Chen et al., 2015; Foster et al., 2017) consist of drug delivery systems conjugated with carriers, ligands or antibodies to selectively concentrate drugs in target organs, tissues, cells, or intracellular structures through local, oral or systemic administration. At present, the targeted delivery of nanoparticles can be achieved by active and passive targeting. Passive targeting (Meng et al., 2011; Tang et al., 2013; Tang et al., 2014) is mainly based on the enhanced permeability and retention (EPR) effect. Compared with the microvascular endothelial space in tumor tissue, the microvascular endothelial space in normal tissue is dense, and the structure is complete. Solid tumor tissue is rich in blood vessels and has a large vascular wall surface area, poor structural integrity, and a lack of lymphatic drainage. Such tissue demonstrates selectivity, high permeability and retention of nanoparticles. The EPR effect promotes the selective distribution of macromolecular substances in tumor tissues, which can increase the efficacy and reduce the systemic side effects of drugs. To explore the role of particle size in determining biological characteristics and antitumor activity, Li et al. (Tang et al., 2014) compared nanoparticles 20, 50, and 200 nm in size according to radiolabeling, lung pathology, mathematical modeling and other research results and showed that the 50 nm MSNs performed best in terms of the size-dependent biodistribution, penetration and clearance of tumor tissue, and anticancer efficacy in various tumor models.

Active targeting is achieved through the use of specific targeting ligands, which can enhance specific uptake by cells and increase the intracellular drug concentration. Quan et al. (Quan et al., 2015) designed a drug delivery system called Lac-MSN, in which a 3-aminopropyltriethoxysilane-modified MSN was conjugated with the liver-targeting agent lactose and loaded with the water-insoluble anticancer drug docetaxel (DTX). The system mediated the targeted delivery of asialoglycoprotein receptor (ASGPR) and increased the concentration of the drug in the target organs, resulting in improved bioavailability. Sarkar et al (Sarkar et al., 2016) designed FA-modified quercetin-loaded mesoporous silica nanoparticles (MSNs-FA-Q) targeting breast cancer cells overexpressing folate receptors to enhance cell uptake and improve bioavailability. Lv et al (Lv et al., 2017) modified the folate MSN drug delivery system to achieve active targeting, thus increasing both the drug concentration in the target organ and the therapeutic efficacy.

MSNs allow high loading of drugs, and protects them inside the channeled pores until they reach target and release. The MSNs with optimal drug allow potential possess high loading of drugs, sustained and controlled release, and targeted delivery actions. An increasing number of targeted nanodrugs have been modified through diverse preparations to achieve targeted delivery for different needs.

### Tumor Immunotherapy Based on Mesoporous Silica

**FIGURE 4:**
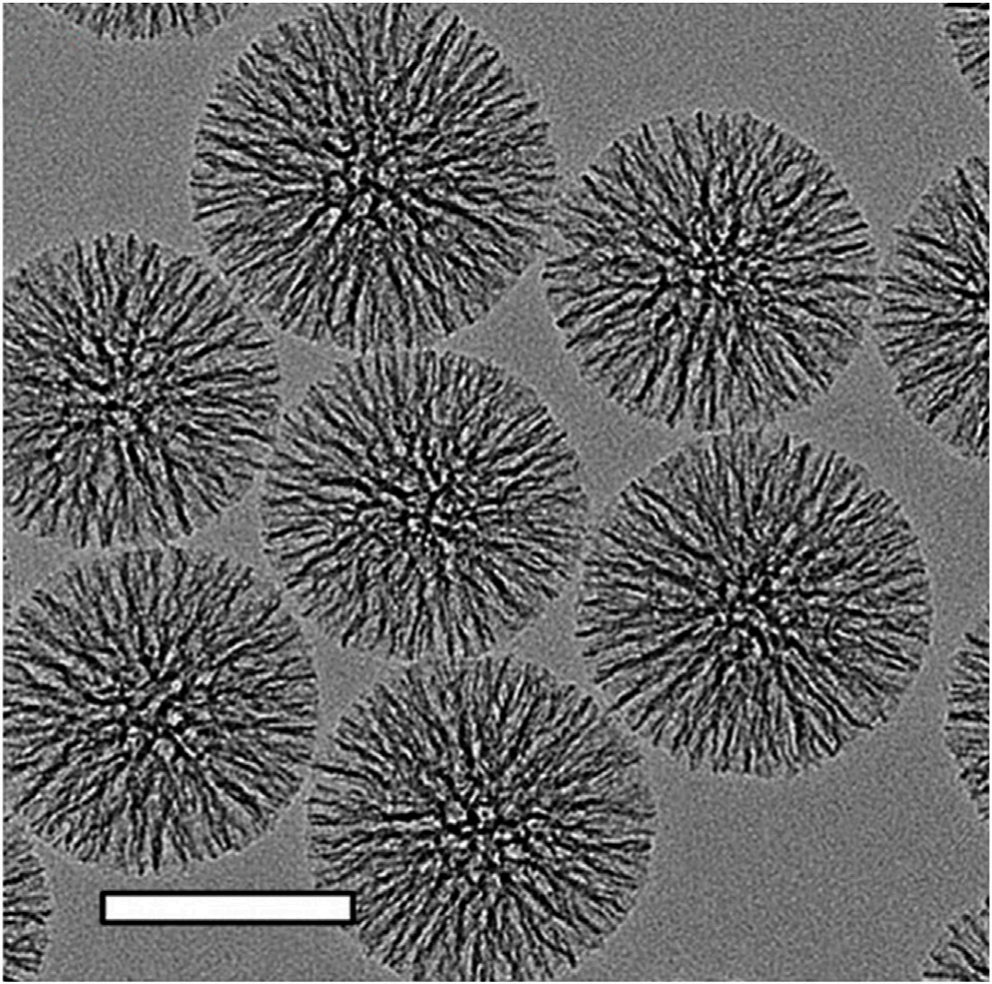
Transmission electron microscopic images of MSNs. The scalebars in TEM images is 100 nm. Reproduced with permission from ref (Shen et al., 2014); copyright 2014 American Chemical Society.

Cancer immunotherapy can be achieved with cancer vaccines, (Nguyen et al., 2020), which are considered to be a promising tool to fight cancer (Figure 4) (Shen D. et al., 2014). Mesoporous silica materials are promising candidates to improve cancer immunotherapy based on their attractive properties that include high specific surface area, high biocompatibility, the easily modified surface groups, and self-adjuvanticity (Lee et al., 2020). Compared with nonporous silica nanoparticles, MSNs not only include self-adjuvanticity, but also could load Toll-like receptor (TLR) agonists, cytosine-phosphonothioate-guanine (CpG), ovalbumin, and immunomodulatory drug (gardiquimod) for mounting a highly potent and long-lasting antitumor immune response. (Nguyen et al., 2019; Nguyen et al., 2020; Seth et al., 2020). Adjuvants (Wang et al., 2012; Liu et al., 2013; Mody et al., 2013) can improve the efficacy of a vaccine by increasing the immunogenicity of the antigen, thus inducing a stronger immune response and promoting the immune memory of the antigen. Liu et al (Liu et al., 2013) designed different structures of silica nanoparticles (SNs) loaded with ovalbumin (OVA) to study the potential for application in protein vaccines. The results showed an effect of the SN structure and size and the medium on protein loading. When mice were vaccinated with SN-OVA preparations, the SNs could upregulate the humoral immune response. Tu et al (Tu et al., 2017) successfully developed lipid-encapsulated MSNPs (LB-MSN-OVA) loaded with OVA as an antigen, which were found to be suitable for the intradermal delivery of encapsulated protein antigens. Modifying nano-microneedles can improve compliance with vaccination, and loading nanoparticles with an antigen can increase and alter the immune response to the antigen. Composite nanomaterials based on mesoporous silica are equally effective in immune delivery systems. Nguyen TL et al (Nguyen et al., 2020) designed an injectable dual-scale mesoporous silica vaccine consisting of coupled mesoporous silica microrods (MSRs) and MSNPs. Compared with the same dose of the MSN vaccine, the MSR-MSN vaccine promoted the production of a large number of cytotoxic antigen-specific T cells against cancer. Compared with the MSR vaccine, the MSR-MSN vaccine showed enhanced antitumor efficacy. The enhanced efficacy of the dual-scale nanoparticle cancer vaccine is due to the improvement in the uptake of MSNs by dendritic cells (DCs) and the migration of the MSNs to lymph nodes over time.

### Assisted Imaging Technology Based on Mesoporous Silica

The use of mesoporous silica in auxiliary imaging technology has made good progress (Caltagirone et al., 2015) and can be divided into three categories: 1) fluorescent agents for fluorescence imaging; (Rashidi et al., 2017); 2. loaded contrast agents for magnetic resonance imaging (MRI); (Cao et al., 2015); and 3) radiolabeled MSNs for positron emission tomography (PET) imaging (Ni et al., 2018).

The MSN that is used in fluorescence imaging is optically transparent due to its nanoscale particle size (Koenderink et al., 2001) and will not interfere with the emission of fluorescent agents. At present, imaging agents based on mesoporous silica mainly include quantum dots (QDs), (Li et al., 2019), organic dyes (Rashidi et al., 2016; Rashidi et al., 2017; Fulaz et al., 2019) or rare-earth elements (Chu et al., 2019). Li et al (Li et al., 2019) designed Ag2Se QD-modified MSNPs. Ag2Se QDs have near-infrared fluorescence, excellent biocompatibility and good dispersibility. Relying on the remarkable photothermal conversion rate (approximately 27.8%) of Ag2Se QDs and the anticancer drug DOX to achieve the effect of combined treatment, responsive multifunctional composite nanomaterials that release drugs at specific sites can achieve effective and accurate cancer treatment. Duan et al (Duan et al., 2017) designed a multifunctional imaging-guided MSN-based therapeutic platform for combined fluorescence/photoacoustic (PA)/computed tomography (CT) imaging and gene/chemical/photothermal therapy. The X-ray opacity and PA properties of AuNRs were used for CT and PA imaging; the fluorescent QDs further improved the imaging sensitivity; and DOX and therapeutic pDNA were used to achieve collaborative imaging-guided cancer treatment.

MRI (Taylor et al., 2008; Cao et al., 2015) is an effective biomedical tool that has the ability to acquire anatomical and metabolic/functional information with high spatiotemporal resolution nondestructively, and MSN-based MRI contrast agents exhibit strong relaxation, high sensitivity and a highly active core payload (Pálmai et al., 2017) due to their flexibility and large surface area. MSNs modified by targeting ligands can effectively identify abnormal tissues for diagnostic purposes. The accumulation of MSN-based MR contrast agent at the target site helps to improve imaging sensitivity. Marcell et al. (Pálmai et al., 2017) designed a silica-based MRI contrast agent with a shortened longitudinal (T1) relaxation time due to the presence of Mn^2+^. The functionalized MSNs showed significant contrast enhancement on MRI both *in vivo* and *in vitro*. Similarly, Li et al. (Li et al., 2018) designed a mesoporous silica-based T1-T2 dual-core contrast agent loaded with Fe_3_O_4_ and BSA-Gd2O3 nanoparticles. It has been verified that T1-T2 dual-mode MRI avoids the false-positive signals observed in single-mode imaging and provides more accurate and complementary information.

PET imaging (Ni et al., 2018) of radiolabeled SiNPs is used for analysis of the distribution of drugs in organs/tissues, pharmacokinetic determination and tumor targeting monitoring and has the advantages of high sensitivity, noninvasiveness and quantitation. The U.S. FDA approved the use of 124 I-labeled ultrasmall SiNPs (C dots) for PET imaging in patients with metastatic melanoma as an investigational new drug (IND), which is a significant milestone for the development of useful mesoporous silica products. At present, MSNPs labeled with different radioactive elements (64Cu, 89Zr, 18F, 68Ga, 124I, etc.) have been developed for cancer treatment.

Clinically, the new technology of combined PET/MRI imaging (Forte et al., 2019) has demonstrated high sensitivity and good accuracy and has value in the early detection and diagnosis of many diseases (especially tumors and the most common heart and brain diseases). In the future, imaging technology will continue to move toward integration and development, which will increase the requirements for imaging agents based on mesoporous silica.

### Other Medical Applications

MSNs have great potential for applications in other biomedical fields due to their excellent properties. MSNs loaded with antibiotics and bone cement for functional application in orthopedic surgery have excellent mechanical properties and sustainable drug delivery efficiency (Shen et al., 2011; Letchmanan et al., 2017). A large number of studies have shown that such materials can efficiently and continuously release antibiotics, thereby reducing the risk of postoperative joint infections. Recently, Hiroshi Ikeda et al (Ikeda et al., 2019) designed a mesoporous silica-based organic-inorganic composite material with a nanoscale double-mesh structure to achieve the mechanical properties of human enamel, and the material demonstrated hardness compatible with that of human enamel. Furthermore, Li et al (Li et al., 2015) developed a removable denture base or orthodontic appliance loaded antibiotics to achieve a continuous sterilization effect.

## Biocompatibility and Biodistribution

**FIGURE 5:**
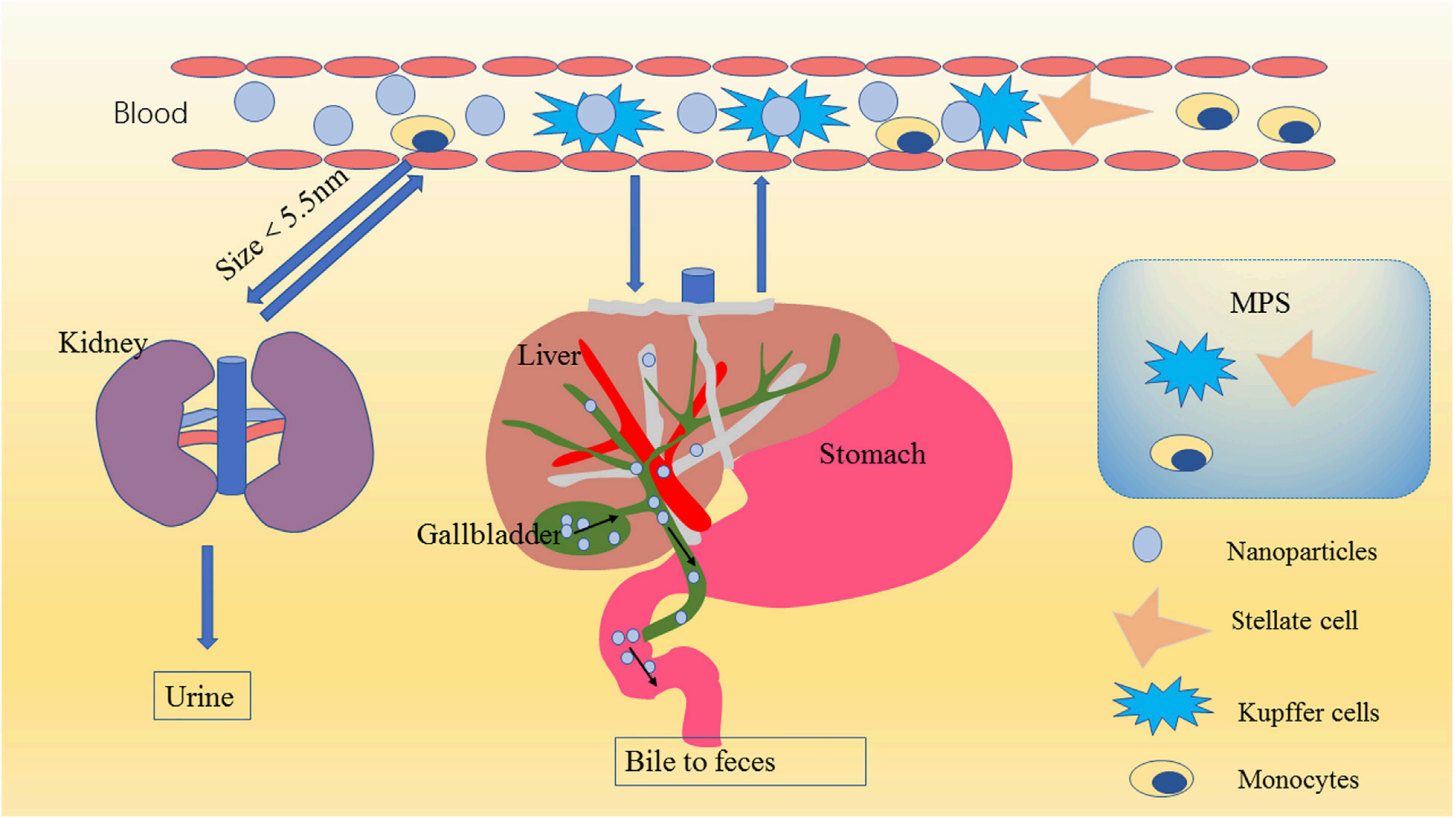
Schematic diagram of nanoparticle metabolism. MSNs in the blood are eliminated mainly through the kidneys (urine) or liver (bile to feces) after encountering the mononuclear phagocyte system (MPS). Because of the filtration effect of the glomerulus and because the physiological pore size of the glomerulus is approximately 5.5 nm, MSNs smaller than 5.5 nm are excreted mainly in the urine through the kidney; MSNs larger than 5.5 nm transition from the peripheral circulation to the liver, and the nanomaterials that escape through the liver return to the systemic circulation through the central vein and are finally returned to the liver (or another MPS organ). This process is repeated until the nanomaterial is removed from the blood. The liver degrades the MSNs into various types of silicic acid, which are excreted in the urine through the hepatic vein to the kidney, usually within 4 weeks. The undegraded MSNs enter the common bile duct, are excreted to the duodenum through the sphincter of Oddi, and finally pass through the entire gastrointestinal tract to be cleared in the feces.

The safety of MSNs is very important. We summarized the metabolic process of mesoporous silica in the body, which is of great significance for its biosafety and biocompatibility, according to a large amount of literature (Figure 5); (Fischer et al., 2006; He et al., 2011; Fu et al., 2013)

Drugs based on liposome nanocarriers have been used in clinical treatment; (Bozzuto and Molinari, 2015; Rudokas et al., 2016); current clinical data show that these carriers are sufficiently safe, possibly due to the biocompatibility of phospholipids (Allen and Cullis, 2013; Palazzolo et al., 2018). Similarly, polylactic acid-glycolic acid copolymer is a biodegradable functional organic polymer that can be used as a polymeric nanocarrier (Kamaly et al., 2012; Palazzolo et al., 2018) with good biocompatibility, no toxicity, good encapsulation and easy formation. Due to its performance, this material has been approved by the FDA and officially included in the United States Pharmacopeia as a pharmaceutical excipient. Although these nanocarriers have demonstrated satisfactory biocompatibility, they still have some shortcomings, such as problems related to stability, controlled drug release, and biological barriers. However, MSNs show good performance in these aspects. In this section, we focus on the progress in research on MSN biodistribution and biocompatibility.

### Influence of MSN Shape, Size and Surface Modification

The biosafety of MSNs is related to their surface modification. Studies have shown that the cytotoxicity of MSNs is mainly related to their surface silanol groups, which may nonspecifically bind to certain proteins on the cell membrane and cause cell lysis and necrosis (Slowing et al., 2009; Lin and Haynes, 2010; Shinto et al., 2019; Tsamesidis et al., 2020). The surface properties of MSNs have an impact on their biodistribution and biocompatibility. Researchers have found that using polyethylene glycol (PEG) for functional modification to change the surface characteristics of MSNs can reduce the capture of MSNs by the liver, spleen, and lungs, thereby prolonging their circulation time (He et al., 2011). Breznan et al (Breznan et al., 2018) designed a series of MSNs of different sizes (25, 70, 100, 170 and 600 nm) and with different surface features (pristine, C_3_-COOH moieties and C_11_-COOH moieties) to study their cytotoxicity and inflammatory potential. *In vitro* and *in vivo* experiments showed that the toxicity of mesoporous silica was lower than that of nonporous nanoparticles and that changing the surface charge of MSNs can further reduce their cytotoxicity. Further analysis showed that particle size, zeta potential and surface modification were important factors affecting MSN cytotoxicity. Yu et al.'s (Yu et al., 2011) cytotoxicity and hemolysis test results for MSNs also support this conclusion. Roggers et al.(Roggers et al., 2014) found that lipid bilayer-coated MSNs showed significantly better blood compatibility than phosphatidylcholine-coated and uncoated MSNs. Through electron microscopy, ultraviolet-visible spectroscopy and flow cytometry analysis, it was observed that these inorganic/organic composite nanomaterials could contact red blood cells without damaging the cells even at relatively high concentrations. This finding also reflects that changing the surface properties of MSNs through different surface modification strategies can improve their biocompatibility. Liu et al. (Liu et al., 2011) showed that in a mouse model, the lethal dose of 110 nm MSNs was higher than 1,000 mg/kg. Repeated dose toxicity studies showed that no deaths occurred within 14 days of the intravenous administration of repeated MSN doses of 20, 40 and 80 mg/kg. These results also indicated that the toxicity of MSNs is lower when administered via a single-dose intravenous injection than when administered repeatedly. These data show good biocompatibility.

To explore the effects of particle size, dose and cell type on MSN biodistribution and toxicity, Kim et al.(Kim et al., 2015) synthesized MSNs with particle sizes of 20, 60, 100, and 200 nm and cultured them with A549 human alveolar cancer cells and HepG2 lung cancer cells. In addition, in NIH/3T3 mouse embryonic fibroblasts, MSNs of a uniform particle size, to eliminate the experimental uncertainty caused by mixed particle size, were used to explore the effect of particle size on cytotoxicity. By analyzing cell viability, membrane destruction, oxidative stress, and cell uptake, this research showed that MSN cytotoxicity was closely related to particle size, cell type and MSN dose.

The biodistribution and biological toxicity of MSNPs are closely related to their physical and chemical properties. While surface modifications have the greatest influence, different surface modifications have different degrees of influence. The size of the nanoparticle, the surface charge, surface modification, geometric shape and biodistribution are closely related to the biodistribution and biological toxicity. This suggests that when designing nanoparticles, the size and surface modifications should be selected according to the drug delivery strategy and treatment requirements.

### Biodistribution of MSNs

To obtain the best therapeutic effect, nanoparticles should accumulate in diseased tissue as much as possible while having no adverse effects on normal tissue. Upon completing the task of drug delivery, nanoparticles should be cleared from the body and nontarget sites and not persist in the organism.(Pérez-Herrero and Fernández-Medarde, 2015). Although smaller nanoparticles exhibit stronger tissue penetration, they cannot distinguish between healthy and diseased tissues.(Heldin et al., 2004).

At present, there are six main methods for administering nanoparticles: intravenous injection; subcutaneous injection; intratumoral injection; pulmonary inhalation; oral administration; and transdermal administration. Intravenous injection can achieve the rapid delivery and distribution of nanoparticles in the circulatory system, but it can also cause them to be cleared by the kidneys, liver or reticuloendothelial system (RES). The advantage of intravenous injection, subcutaneous injection and intratumoral administration is that these methods can overcome the “first-pass effect” of the liver; after subcutaneous injection, according to the size of the nanoparticles, large nanoparticles (tens of nanometers) enter the lymphatic circulation, and small nanoparticles (a few nanometers) enter the blood circulation through the capillaries. It has been shown that larger materials can last longer at the injection site, and intertumoral injection has been the most common method used in animal experiments; (Brigger et al., 2002; Heldin et al., 2004); this method can achieve more precise targeting and overcome the limitations of systemic administration. Pulmonary inhalation (Gehr et al., 1978; Patton and Byron, 2007) is a feasible method for treating lung diseases or for systemic delivery to treat other diseases because the lungs (alveoli and airways) have a large internal surface area and high endothelial permeability. Nanomaterials that reach the alveolar exchange area are cleared by alveolar macrophages (phagocytes that may leave or remain in the lungs) or DCs and then transported to the draining lymph nodes of the lungs. The oral administration of nanomedicine (Desai et al., 2012) is relatively convenient and has high patient compliance. However, in enzymatic degradation in acidic environments, the intestinal tissue barrier and the “first-pass effect” of the liver (Zhang YN. et al., 2016) all lead to poor oral bioavailability.(Yun et al., 2013). Additionally, healthy human skin is an effective barrier to nanoparticles.(Lodén et al., 2011). Systemic treatment though transdermal delivery is therefore unlikely, but the use of this route for the treatment of local diseases (such as psoriasis) is still promising.(Boakye et al., 2017).

The physical and chemical properties of nanomaterials affect not only the biological distribution but also the biodegradation rate of the nanomaterials in the body. The biodistribution of nanomaterials is related to many factors, including the size, surface properties and dissolution rate of the nanomaterials, as well as tissue- or organ-dependent factors, such as tissue permeability and barrier properties. After intravenous, oral and subcutaneous delivery, nanomaterials can be found in the liver, spleen, kidneys, bone marrow, central nervous system, and local and systemic lymph nodes, (Alexis et al., 2008; Huang et al., 2011; Khlebtsov and Dykman, 2011; Meng et al., 2011), but the liver is the target organ because the liver contains many vascular endothelial cells and Kupffer cells (KCs) and is the main organ for removing macromolecules and microorganisms in the circulation. More than 99% of the particles will be removed by cells in the liver and other organs of the RES. Unexpectedly, when nanomaterials enter and pass through the liver, their speed is reduced by 1,000 times, which increases the possibility of nanomaterials interacting with and subsequently being absorbed and eliminated by a variety of liver cells. These cells are mainly KCs, liver B cells and sinusoidal endothelial cells.(Ray, 2016). He et al. studied the effects of PEGylation and particle size on the biodistribution of MSNs *in vivo*. Nanoparticles 80–390 nm in size were injected into the tail vein of mice and rats. The biodistribution of the nanoparticles was tracked using fluorescein isothiocyanate (FITC), and the results showed higher concentrations of particles in the spleen, liver, and lungs and lower concentrations of particles in the kidneys and heart. The size of nanoparticles also showed a negative correlation with the circulation time. MSNs 80 nm in size showed the longest cycle time, and PEGylation could significantly extend the cycle time. Larger nanoparticles showed faster degradation, which may have been caused by the RES.(He et al., 2011). Xie et al.'s study on the size of MSNs also supports the above conclusion.(Xie et al., 2010). By changing the pore size, geometry and surface characteristics to study the biological distribution, Yu et al. found that the tissue distribution depends on the pore size and surface charge of the particles and that the surface charge and pore size are more important than geometric properties.(Yu et al., 2012).

The biodistribution of MSNPs is closely related to their physical and chemical properties. The size of the nanoparticle greatly affects its biodistribution, and the surface charge, surface modification, geometric shape and biodistribution are closely related to the biodistribution. While surface modifications have the greatest influence, different surface modifications have different degrees of influence. This suggests that when designing nanoparticles, the size and surface modifications should be selected according to the drug delivery strategy and treatment requirements.

### Biological Toxicity of MSNs

Now, we understand the metabolism of MSNs, the factors that affect their metabolism, and their distribution in the body. However, understanding these facets is not sufficient. To realize the clinical transformation of MSNs, only investigating the acute toxicity and subacute toxicity of nanoparticles cannot fully reflect their biological toxicity (Hosseinpour et al., 2020; Mohammapdour and Ghandehari, 2022). It is necessary to understand the toxicity of nanoparticles to various types of cells *in vivo*. At present, scientists have paid attention to the immunotoxicity and cytotoxicity of nanoparticles. However, research on MSN that can cross the blood-brain barrier is still insufficient, because it may damage nerve cells. And the research on the influence of MSNs on DNA is far from enough. In future experiments, more evidence is needed to clarify the biological toxicity of MSN, particularly the following four aspects: immunotoxicity, cytotoxicity, (Manke et al., 2013), neurotoxicity, (Xue et al., 2012), and genetic toxicity.(Donaldson et al., 2010).

Immunotoxicity: The body’s first line of defense includes monocytes and macrophages. Nanoparticles entering the human body interact with immune cells and can cause the production of proinflammatory factors, in turn leading to cell apoptosis, which is considered to be the mechanism of nanoparticle-induced immunotoxicity.(Lee et al., 2011). When the therapeutic doses of nanoparticles are given to patients, what will happen to their immune system? How long does it take for the MSNs to degrade and be cleared from MPS? Weather MPS is saturated or not? And what may happen when MPS is saturated? How does this influence the function of the immune system and the normal functions of saturated macrophages? We can’t ignore these problems.(Shahbazi et al., 2013).

Cytotoxicity: MSNs can induce oxidative stress and mediate apoptosis in a size- and dose-dependent manner through the mitochondrial pathway. ROS-mediated toxicity is considered to be an important mechanism of nanoparticle toxicity.(Fu et al., 2014).

Neurotoxicity: Very small nanoparticles can be transported across the blood-brain barrier and damage nerve cells. Studies have shown that direct contact with neuroblastoma cells induces adverse reactions, including markers of Alzheimer’s disease.(Yang et al., 2014).

Genotoxicity: MSNs can directly interact with DNA, consuming antioxidants, oxidizing DNA, disrupting the cell cycle and causing abnormal gene expression. These are considered to be the potential mechanisms of nanoparticle-induced genotoxicity.(Donaldson et al., 2010; Tarantini et al., 2015).

Currently, it is believed that the main mechanism of biological MSN toxicity is the induction of oxidative stress, which causes toxicity to cells, including immune and nerve cells, as well as DNA. Of course, the toxicity of MSNs is strongly related to the dose, particle size, shape, and surface properties of the MSNs. This is also a research direction that will continue to be explored in the future. For the development of effective mesoporous silica-based clinical drugs, it is necessary to study the potential mechanisms of toxicity.

## Conclusion and Outlook

This review provides a detailed introduction to the applications of MSNs in the field of medicine and how they can improve the bioavailability of drugs, reduce antitumor drug resistance, and facilitate targeted therapy and bioimaging, among others. MSNs have achieved remarkable results in animal experiments. The US FDA has also approved the first silica material, C dots, for clinical research. Porous silica holds great promise. Scientists have conducted much research to develop MSNs and apply them as drug delivery systems or in therapeutic strategies. The biodistribution and long-term fate of these MSNs are critical to the applicability and efficacy of drugs, and gaining an understanding of these outcomes is essential to promote the clinical application of mesoporous silica.

In the development of drug delivery strategies, scientists continue to make advances in synthesis methods, surface modifications, and targeting strategies. Composite materials and multifunctional materials that can achieve active targeting and bioimaging while delivering drugs are constantly emerging. In oncology, better therapeutic effects can be achieved through the combination loading of pharmaceuticals and genetic vectors, with the potential to result in a full response. Now, not only reactive oxygen and acid-base responses but also precision delivery under external conditions can be achieved via controlled release in response to photonic, ultrasonic, or magnetic stimuli. In future research, the development of multifunctional responsive materials through different targeting and response strategies and in combination with various imaging technologies will lead to the efficient treatment of target tissues with reduced side effects.

Currently, research on the biodistribution and biosafety of mesoporous silica is very important for its application as a nanocarrier. Studying the biological toxicity of MSNs, particularly their immunotoxicity, cytotoxicity, neurotoxicity and genotoxicity, is important for achieving the clinical application of MSNs.
